# The Prince

**Published:** 1889-04-27

**Authors:** 


THE ROYAL NUMBER OF

### The Hospital

A WEEKLY INSTITUTIONAL JOURNAL OF

Science, Medicine, Nursing, and Philanthropy.

*For Hospitals, Asylums, Medical Practitioners, Students, Nurses, Families, Parishes, Congregations, and the Charitable Public.*

VOL. VI. No. 135.] [APRIL 27, 1889.

### Prince, Princess, and People.

### THE PRINCE.

PRINCES are to be compared with princes, and peasants with peasants. There must be those that answer to princes and peasants in every conceivable condition of society. Loyalty, which means honesty of heart, is as indispensable in the true prince as in the people; and, if there can be degrees of obligation in that which is imperative upon all men, the loyalty should rise with the exaltation of the man, and should find its flower and perfect fruit on the throne. The histories of this and of other countries preserve the memories of monarchs of whom it is not desirable to speak in this place. But there are names inscribed on the page of time which will never fade from the grateful recollection of man. Space does not permit even of the mention of more than one or two. At this moment Britain is under the sway of a Queen; and it is natural to place her in comparison, and perhaps in contrast, with queens who have occupied the throne before her. The only English queen who may properly be compared with the present monarch is Queen Elizabeth. She made the name of England feared and honoured throughout the world. If Britain is less feared in some parts of the world to-day than in Elizabeth's time she is more honoured. The name of Queen Victoria is the synonym for all that is queenly and womanly wherever it is known.

Times have changed since Elizabeth, a heroic figure, put courage into the hearts of her ministers, and enthusiasm into the souls of her people. Those were fighting days. That state and that monarch which feared to fight might not hope to live. Elizabeth did not fear to fight, and she was mistress of a people which has at all times been willing to die in the cause of a brave and loyal leader. Many were the faults of the maiden queen; but he would be less than an Englishman who did not recognise that her faults were as nothing compared with the more than manly courage that fired her breast from the first day of her reign to the last. We admire Elizabeth: Victoria we admire and love. Not since the commencement of her reign has this country ever been called upon to fight for its life. Waterloo had been fought before she was born, and the victory of the allies was the stroke of fate which gave to England a long and welcome peace. We can still fight, if need be, and the enthusiasm which kindled the nation in the days of Elizabeth would still rise higher in these of Queen Victoria if occasion should demand. But other and worthier passions than those of war animate the majority of the people, and the Queen, with a sympathy as profound as it is noble, encourages the arts of peace and the growth of universal brotherhood and goodwill. To her, and to the Prince Consort, of great and virtuous memory, this country owes a debt of gratitude, deep, lasting, and immeasurable.

The Prince of Wales, whose public work in connection with charity is the inspiration of our present number, was born of parents loyal to the heart's core to the best interests of the people. From his earliest days he has been taught to see his duty and his pleasure in the furtherance of their well-being. Circumstances did not call him to the battle-field, happily for us;

ARMY MEDICAL LIBRARY

but they did point him to a field of duty, less glorious, indeed, from the point of view of him who loves pomp and circumstance, the beat of the drum, and the shouts of those that conquer, but far more human, far more gentle, far more noble, and far more enduringly worthy than any battle-field, except that which is trodden by the heroes who defend our native soil. The Prince has always shown himself admirably fitted for the part he has to play. That he has succeeded so well in this is no evidence that he would not equally well have played a different part if it had been his duty to do so; rather is it a proof to the contrary. He who can do well the particular duty that falls to his lot, thereby gives presumptive proof that he could adapt himself to other positions and duties if circumstances demanded it.

The central point in the Prince's character which commends him to the regard of Britons, and which no man can dispute, is his robust good sense and undoubted kindness of heart. Few men have more correctly taken the measure of his fellow-countrymen than he, and still fewer princes. If there is any kind of person that our countrymen cannot tolerate in high places it is the fool, the man who has no understanding of and no sympathy with the peculiar characteristics of the people among whom he is placed. Princes love obedience to prescribed authority and reverence for law. Thus they themselves, being so conspicuously placed, should show their own reverence for that which is the Divine limitation within which every man must move. That is exactly what the Prince has done. He has made no foolish claims upon the forbearance of the people in this respect, but has shown himself a man well disposed to regard with equal eye the just rights of all with whom he has come into relation. Men of every conceivable shade of political opinion are all agreed as to this, and especially those who have had any degree of personal knowledge of the Prince. It is wonderful what personal contact can accomplish in the way of removing misunderstandings. The Prince of Wales, if ever any man did, has lived among the people, and made himself the genial and kindly friend of all. Philanthropy is the special sphere of this journal; philanthropy has been, perhaps, the special sphere of the Prince of Wales from his boyhood. May it not be said, with the most undoubted certainty, that every genuinely good cause in the kingdom for which his aid has been sought, and which it has been possible for him to help, has received that aid in full measure? Charities of every name and kind have had from him the best encouragement he could give. Every man of experience in the charitable world has long known full well that to secure the name of the Prince of Wales for any movement it was enough to show that the movement was good and useful and of real public importance. Merely to enumerate the philanthropic and useful movements he is connected with would more than fill the space at our disposal. Hospitals, orphanages, convalescent homes, schools for the poor, for the better classes, technical schools, colleges of music, everything that helps to relieve sickness, ameliorate poverty, improve the lot or brighten the life of the people, has found in the Prince of Wales a willing patron and a warm friend. These things it is well to consider. In the strife of the times and the struggle for existence we sometimes forget the sunnier side of national life, and think only of the present moment of gloom and difficulty. Venerable institutions, when worthily maintained, add greatly to the security as well as to the dignity of the state and the people. No man can be too thankful, whatever his politics, when institutions, good in themselves, are made strong and permanent by the worthiness of those who administer them. To Queen Victoria and to the Prince of Wales is largely due that ordered security and solid prosperity which we as a nation so largely enjoy. Whatever undisciplined youths and reckless iconoclasts may think and say, every man of sober years and mature judgment will echo with grateful enthusiasm the song of the children,

"God bless the Prince of Wales."

---

